# Hsp90 inhibition ameliorates CD4^+^ T cell‐mediated acute Graft versus Host disease in mice

**DOI:** 10.1002/iid3.127

**Published:** 2016-10-10

**Authors:** Carsten Berges, Thomas Kerkau, Sandra Werner, Nelli Wolf, Nadine Winter, Thomas Hünig, Hermann Einsele, Max S. Topp, Niklas Beyersdorf

**Affiliations:** ^1^ Department of Internal Medicine IIDivision of HematologyUniversity Hospital WürzburgWürzburgGermany; ^2^ Institute for Virology and ImmunobiologyUniversity of WürzburgWürzburgGermany

**Keywords:** Acute graft‐versus‐host disease, CD4^+^ T cells, graft versus tumor, heat shock protein 90, hematopoietic stem cell transplantation, regulatory T cells

## Abstract

**Introduction:**

For many patients with leukemia only allogeneic bone marrow transplantion provides a chance of cure. Co‐transplanted mature donor T cells mediate the desired Graft versus Tumor (GvT) effect required to destroy residual leukemic cells. The donor T cells very often, however, also attack healthy tissue of the patient inducing acute Graft versus Host Disease (aGvHD)—a potentially life‐threatening complication.

**Methods:**

Therefore, we used the well established C57BL/6 into BALB/c mouse aGvHD model to evaluate whether pharmacological inhibition of heat shock protein 90 (Hsp90) would protect the mice from aGvHD.

**Results:**

Treatment of the BALB/c recipient mice from day 0 to +2 after allogeneic CD4^+^ T cell transplantation with the Hsp90 inhibitor 17‐(dimethylaminoethylamino)‐17‐demethoxygeldanamycin (DMAG) partially protected the mice from aGvHD. DMAG treatment was, however, insufficient to prolong overall survival of leukemia‐bearing mice after transplantation of allogeneic CD4^+^ and CD8^+^ T cells. Ex vivo analyses and in vitro experiments revealed that DMAG primarily inhibits conventional CD4^+^ T cells with a relative resistance of CD4^+^ regulatory and CD8^+^ T cells toward Hsp90 inhibition.

**Conclusions:**

Our data, thus, suggest that Hsp90 inhibition might constitute a novel approach to reduce aGvHD in patients without abrogating the desired GvT effect.

## Introduction

Acute Graft versus Host Disease (aGvHD) is still a major complication following allogeneic bone marrow or hematopoietic stem cell (HSC) plus T cell transplantions, which are most widely used for leukemia treatment 1, 2. It is the transplanted allogeneic T cells contained in the graft which on the one hand induce aGvHD, but on the other mediate the Graft versus Tumor (GvT) effect—required for successful eradication of leukemia—enhance HSC engraftment and also protect the patient from infectious complications 1, 3, 4. Therefore, very promising novel therapeutic concepts, transfering CD4^+^Foxp3^+^ regulatory T cells (Tregs), achieve protection from aGvHD, while sparing the beneficial effects of the allogeneic T cells, most importantly the GvT effect 5, 6, 7, 8, 9, 10, 11. Tregs enriched by sorting ex vivo protect the recipient from aGvHD. This has first been shown to be effective in mouse models of aGvHD, but specialized centers are now starting to make adoptive Treg transfers, also post stem cell transplant, available for patients 12, 13, 14. Alternatively, selective therapeutic expansion and activation of Tregs versus conventional CD4^+^ and CD8^+^ T cells also protects from aGvHD. Here, anti‐CD28 mAb, either ligand binding‐blocking 15 or superagonistic 10, 11, have been used successfully in animal models. In patients, low dose Interleukin(IL)‐2 treatment has been shown to expand Tregs and to ameliorate symptoms in chronic GvHD 16, 17, 18. In addition, treatment with rapamycin, which blocks the mammalian, now mechanistic target of rapamycin (mTOR), preferentially inhibited conventional CD4^+^ and CD8^+^ T cells over Tregs protecting mice from aGvHD 19. In contrast to enriching unmanipulated Tregs ex vivo 6, 7, 8, 9 or to expand them with superagonistic anti‐CD28 mAb in vivo 10, rapamycin treatment abolished the GvT effect in the mouse model 20. As the Akt/mTOR axis appears ideal for differential targeting of conventional CD4^+^ T cells versus Tregs due to the known rapamycin resistance of mouse 19 and human Tregs 21 drugs other than rapamycin interfering with this signaling pathway might show a more favourable profile. One such class of drugs are Heat shock protein (Hsp) 90 inhibitors preventing chaperoning of client proteins like Akt by Hsp90 22, 23. In the absence of Hsp90 binding, the client proteins easily become targets of proteasomal degradation 22. It has recently been shown for human T cells in vitro that Hsp90 inhibition by 17‐(dimethylaminoethylamino)‐17‐demethoxygeldanamycin (DMAG) induces apoptosis in activated T cells while sparing resting T cells 24. Very recently, the water insoluble DMAG analogue 17‐allylamino‐17‐demethoxygeldanamycin has been shown to fortify the gut epithelium against the attack of allogeneic T cells partially protecting mice from aGvHD in vivo 25.

In this study, we evaluated the efficacy of Hsp90 inhibition by DMAG in the C57BL/6 into BALB/c mouse model of aGvHD. Apart from aGvHD we also studied the GvT effect against the BCL_1_ lymphoma and performed a series of in vitro experiments to disect the responsiveness of CD4^+^ and CD8^+^ T cells toward Hsp90 inhibition. Our results suggest that Hsp90 preferentially targets conventional CD4^+^ T cells over both Tregs and CD8^+^ T cells. In consequence, Hsp90 inhibition partially protected the mice from aGvHD while not abrogating the wanted GvT effect.

## Material and Methods

### Animals

CD90.1‐congenic C57BL/6 mice were bred at the animal facility of the Institute for Virology and Immunobiology, University of Würzburg. C57BL/6.OlaHsd BM donors and BALB/c.OlaHsd hosts for aGvHD experiments were obtained from Harlan–Winkelmann. C57BL/6 and C57BL/6.CD90.1‐congenic mice were used for experiments between 6 and 12 weeks of age. BALB/c mice were irradiated at the age of 9–10 weeks. All experiments were performed according to German law and approved by the Government of Lower Franconia as the responsible authority.

### Reagents and antibodies

17‐(dimethylaminoethylamino)‐17‐demethoxygeldanamycin (DMAG) was purchased from Invivogen and NVP‐AUY922 (AUY) were bought from Selleckchem. Primary monoclonal (mAb) antibodies were obtained from the following sources: Purified anti‐mouse CD3, Alexa647‐conjugated anti‐mouse CD4, PerCP‐ or eFluor 450‐conjugated anti‐mouse CD8, FITC (fluorescein isothiocyanate)‐conjugated anti‐mouse I‐A^b^, biotin‐conjugated anti‐mouse CD90.1, PE‐conjugated anti‐mouse/human Helios, APC (Allophycocyanine)‐conjugated Annexin V: Biolegend (London, UK); Purified anti‐mouse CD95, APC‐conjugated anti‐mouse CD107a, PE‐conjugated anti‐mouse Granzyme B, biotin‐conjugated anti‐mouse I‐A^b^; Purified anti‐mouse TCR‐β: BD biosciences (Heidelberg, Germany); PE‐conjugated anti‐mouse CD4, eFluor 450‐conjugated anti‐mouse CD8, PE‐Cy5‐conjugated anti‐mouse Foxp3: eBiosciences (Frankfurt/Main, Germany).

### Fluorescence‐activated cell sorting (FACS)

Up to 10^6^ cells were stained in 50 μl PBS/0.2% BSA/0.02% sodium acide. For blocking of FcγRII/RIII receptors, cells were incubated for 15 min at 4°C with saturating amounts of cell culture supernatant of the clone 2.4G2. Then, cells were stained with fluorochrome‐conjugated or biotinylated mAbs for 15 min at 4°C. Biotinylated antibodies were detected by incubation with either PerCP‐ or APC‐Cy7‐conjugated streptavidin (Biolegend). Then cells were analyzed on a FACSCanto II flow cytometer with the use of the FACS Diva software (BD).

For intracellular staining of Foxp3, cells were first stained with antibodies against surface markers followed by fixation in Fix/Perm buffer (eBiosciences) for at least 30 min at RT. Fixed cells were washed thrice in Perm buffer (eBiosciences) and then probed with antibodies against Foxp3. After additional washing steps with Perm buffer, cells were resuspended in PBS/0.2% BSA) and analyzed by flow cytometry. The following gating strategy was used to analyze the data: First live cells were gated based on forward and side scatter. The live gate was further analyzed for cell surface expression of Thy1.1 and CD4, selecting only Thy1.1^+^CD4^+^ cells (donor T cells). Subsequently, CFSE dilution, surface exposure of phosphatidylserine (Annexin V) and intracellular expression of Foxp3 were determined. For further analyses of the data FlowJo software (FlowJo LLC) was employed.

### aGvHD experiments and tracking of alloreactive T cell responses in vivo

BALB/c mice were conditioned for transplantation by total body irradiation with 8 Gy as a single dose. The mice were assigned to the different experimental groups so that the average body weight before irradiation was about the same in all groups. To reduce the gut flora animals were given Neomycin (250 μg/ml) and Polymyxin B Sulfate (3 U/ml; Sigma–Aldrich, Schnelldorf, Germany) in drinking water, starting 3 days before irradiation until day 28 after transplantation. Approximately 24 h after irradiation the mice received 10^7^ T cell‐depleted bone marrow (TCD BM) cells from C57BL/6 mice and either 5 × 10^4^ or 5 × 10^5^ CD4^+^ T cells or total CD4^+^ and CD8^+^ T cells intravenously. To obtain TCD BM cells, erythrocytes were lysed from total BM preparations by incubation with TAC buffer (20 mM Tris, 155 mM NH_4_Cl, pH 7.2, 10 min, room temperature), then FcRs were blocked with 20 μg/mL normal mouse Ig (Sigma–Aldrich) before T cells were depleted using magnetic‐activated cell sorting (MACS) anti‐CD90.2 beads (Miltenyi Biotec, Bergisch‐Gladbach, Germany) and MACS columns according to the manufacturer's instructions. T cell depletion was approximately 95% on average. CD4^+^ T cells and total CD4^+^ and CD8^+^ T cells were purified from erythrocyte‐lysed splenocytes with average purities of 90% by negative selection using MACS (Miltenyi). After TCD BM and T cell transfer, mice received intraperitoneal injection of 10 μg DMAG/day, 50 μg AUY/day or solvent only (DMSO) both diluted in PBS/TWEEN® (total volume per injection: 250 μl) from days 0 to +2 post‐BM transplantation.

In short‐term in vivo experiments CD90.1 CD4^+^ T cells were labeled with CFSE (2.5 or 5 μM; MoBiTec) and 6 × 10^6^ CD4^+^ T cells were transferred intravenously. Absolute numbers of donor CD4^+^Foxp3^−^ T cells in secondary lymphoid organs and liver three and six days after transplantation were calculated by multiplying the absolute numbers of viable (trypan blue exclusion) cells per organ with the percentages of cells as determined by FACS analysis.

Observers blinded to the treatment measured body weight and rated the clinical appearance of the animals according to the extended Würzburg mouse aGvHD and lymphoma score (WALS) and animals with severe aGvHD had to be killed for humane reasons as published 26.

### BCL_1_ lymphoma model

Irradiated BALB/c mice were injected with 3 × 10^3^ freshly thawed BCL_1_ lymphoma cells 4 h before transfer of TCD BM cells and of either 5 × 10^4^ to 5 × 10^5^ magnetically purified whole CD4^+^ and CD8^+^ T cells. Whenever possible, splenocytes of humanely killed animals were analyzed for the prevalence of BCL_1_ cells by staining with the anti‐BCL1 idiotype mAb Mc106A5. The lymphoma was rated as “end stage” when both greater than 65% of all splenocytes stained positive with Mc106A5 mAb and the number of BCL_1_ cells per spleen exceeded 1 × 10^7^.

### In vitro experiments

To investigate the effect of Hsp90 inhibition on essential functions of T cells, in vitro experiments were performed using isolated LN cells from BALB/c mice. Cells (1 × 10^6^ cells/ml) were either labeled with CFSE or left unstained and were activated with 0.5 μg/mL anti‐CD3 (clone 145‐2C11, Biolegend) and 30 U/mL IL‐2 (Proleukin®) in RPMI 1640 medium (Lonza, Cologne, Germany) supplemented with 10% heat‐inactivated fetal calf serum, 1 mM sodium pyruvate, nonessential amino acids, 100 U/mL penicillin and 100 μg/mL streptomycin, 30 μM mercaptoethanol, and 2 mM L‐glutamine (all Invitrogen, Darmstadt, Germany) for three days in the presence of different DMAG concentrations or DMSO.

### Statistical analysis

Statistical analyses were performed using Excel© software (version 14.0.7, Microsoft Unterschleißheim, Germany) or GraphPad Prism 4.0c (GraphPad prism Software, Witzenhausen, Germany). We compared groups with either a student's *t*‐test (paired or unpaired) or a Mann–Whitney test (both one‐ or two‐tailed) or, for long‐term survival, a *χ* ^2^ test as indicated in the figure legends. Statistical significance was assumed with *P* < 0.05.

## Results

### Treatment with the Hsp90 inhibitor DMAG partially protects from aGvHD

**Figure 1:**
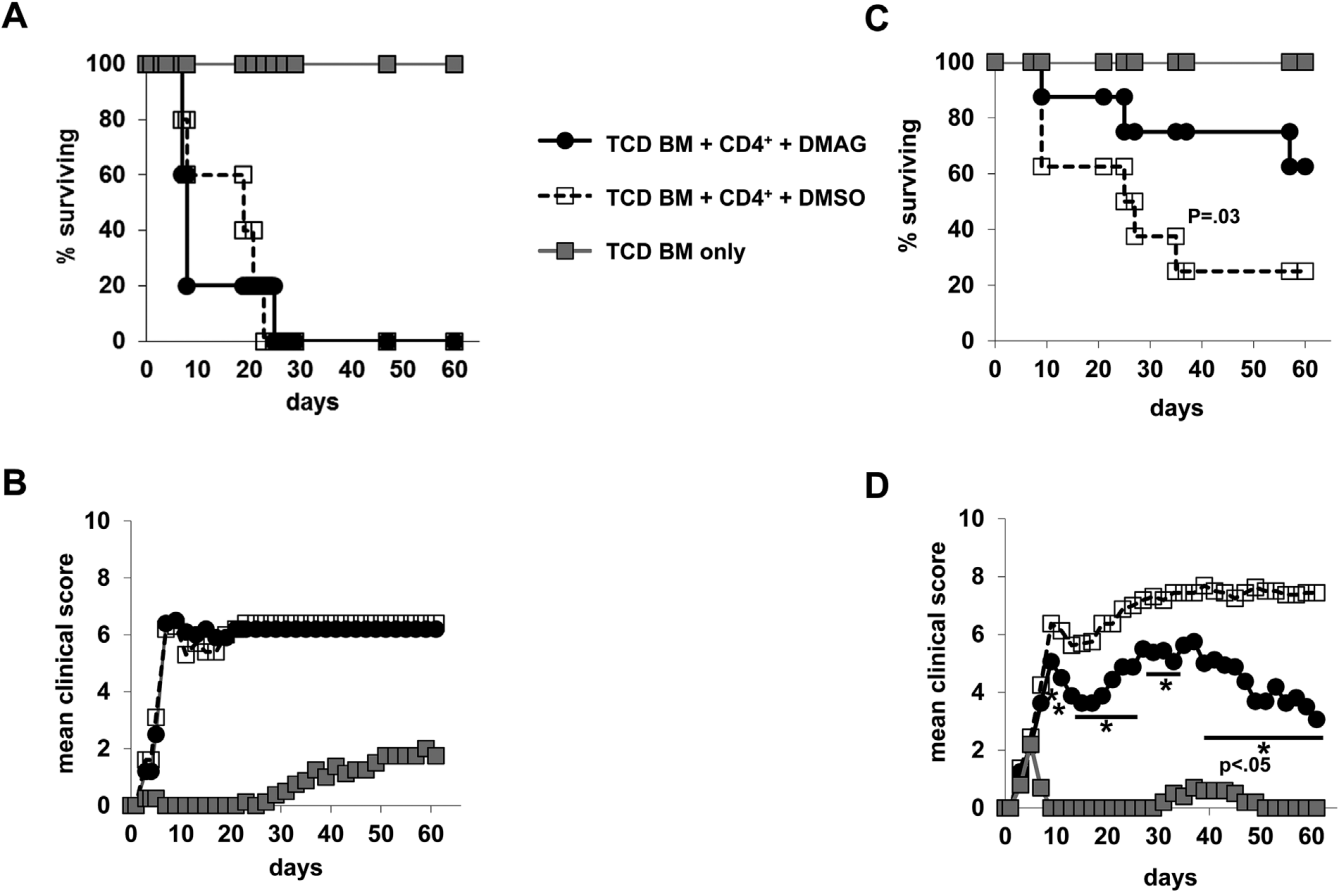
Treatment with the Hsp90 inhibitor DMAG in vivo attenuates CD4^+^ T cell‐mediated aGvHD. Lethally irradiated BALB/c mice were reconstituted with 10^7^ C57BL/6 TCD BM cells either alone (*n* = 5) or with 5 × 10^5^ (A, B) or 5 × 10^4^ (C, D) donor CD4^+^ T cells (*n* = 4–8), respectively. After TCD BM and CD4^+^ T cell transfer, mice received 10 μg DMAG/day or solvent only (DMSO) both diluted in PBS/TWEEN from days 0 to +2 post‐BM transplantation. The percentages of animals surviving over time (A, C) and the mean clinical scores of recipient animals (B, D) are depicted. *P* values refer to the comparison of recipients treated with DMAG versus DMSO only. Data were pooled from two individual experiments. For (C) a *χ* ^2^ test was used and for (D) a one‐tailed Mann–Whitney test.

First, we examined, if Hsp90 inhibition would reduce the severity of aGvHD in vivo. Therefore, we transplanted TCD BM cells from C57BL/6 mice and CD4^+^ T cells from CD90.1‐congenic C57BL/6 mice into lethally irradiated BALB/c recipient mice and treated them immediately, that is, from days 0 to +2 post‐BM transplantation, with the Hsp90 inhibitor DMAG at a dose of 10 μg/day/mouse which is close to the recommended dose for the use in humans 27, 28. Upon transplantation of 5 × 10^5^ CD4^+^ T cells/mouse DMAG treatment did not increase survival (Fig. 1A) or reduce clinical symptoms (Fig. 1B) of aGvHD as compared to mice receiving DMSO only. In contrast, mice receiving 10‐fold less donor CD4^+^ T cells showed improved survival (Fig. 1C) and also reduced clinical severity of aGvHD after DMAG application as compared to the DMSO controls (Fig. 1D). Extending the DMAG treatment beyond day +2 again worsened clinical outcome most likely due to direct toxic effects of prolonged Hsp90 inhibition in the aGvHD target organs (unpublished data). As for DMAG, treatment with the small molecule Hsp90 inhibitor NVP‐AUY922 (AUY, 50 μg/day/mouse) 23 demonstrated no protective effect when mice received 5 × 10^5^ CD4^+^ T cells/mouse (Suppl. Fig. S1A, SB), but reduced clinical symptoms of aGvHD and there was a tendency toward better survival when mice had received 5 × 10^4^ CD4^+^ T cells/mouse (Suppl. Fig. S1C, SD). Thus, Hsp90 inhibition partially protected recipient mice from aGvHD induced by transplantation of allogeneic CD4^+^ T cells.

### Hsp90 inhibition preferentially reduces the accumulation of conventional donor CD4^+^ T cells versus Tregs in vivo

**Figure 2:**
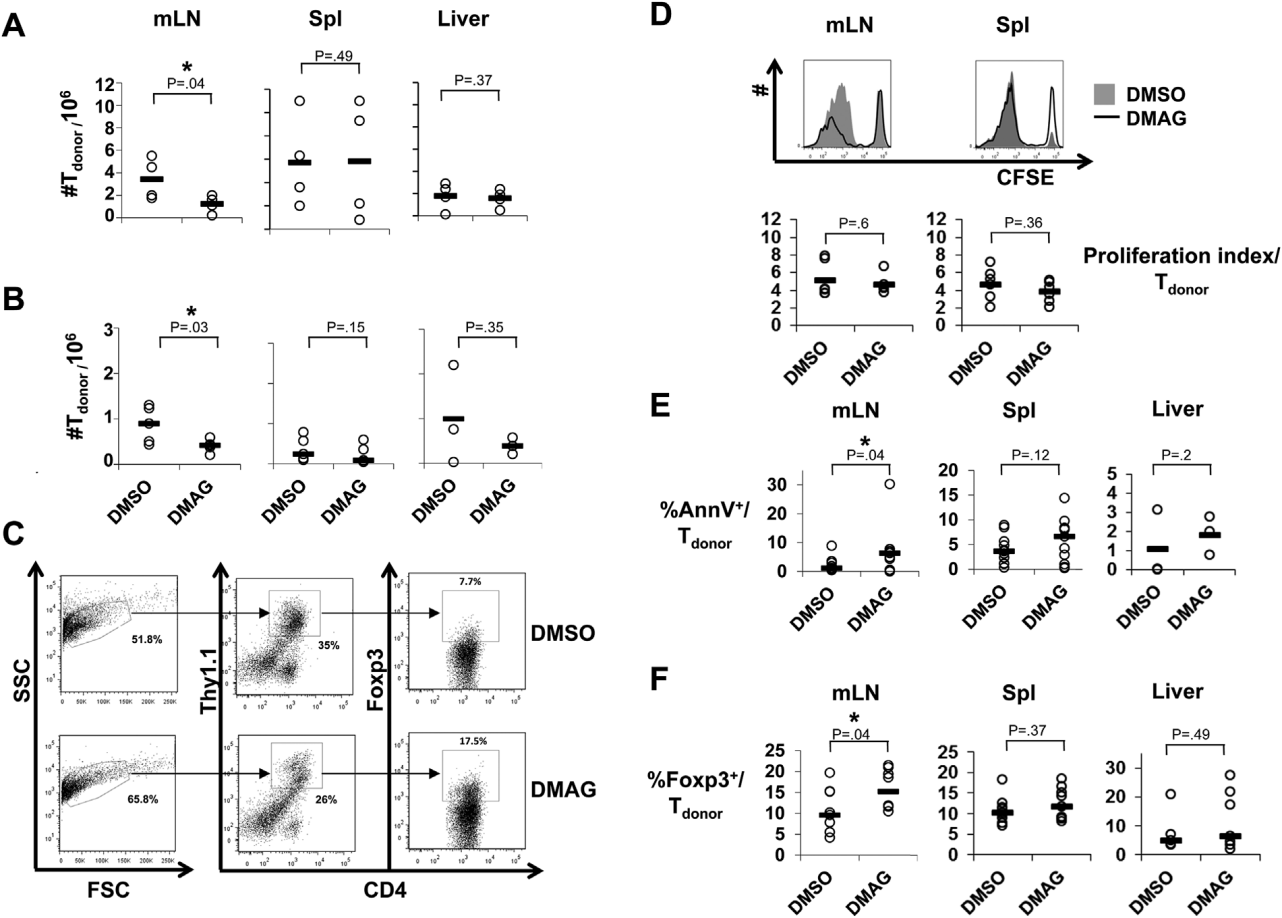
Application of DMAG preferentially impairs expansion of conventional donor CD4^+^ T versus Treg cells in vivo. Donor CD4^+^ T cells were transplanted and mice were treated as in Figure 1. Circles represent individual animals and the horizontal bars the mean values per group. (A, B) Absolute numbers of donor CD4^+^ T cells in mesenteric lymph nodes (mLN, *n* = 4‐5), spleen (Spl, *n* = 4–5) and liver (*n* = 3‐4) seven days after transplantation of 5 × 10^5^ (A) or 5 × 10^4^ (B) donor CD4^+^ T cells (one‐tailed Mann–Whitney test). (C) Gating strategy for flow cytometric analysis of CD4^+^Foxp3^+^ T cells among all donor CD4^+^ T cells in mLN of mice treated either with DMSO (top) or DMAG (bottom). First live cells were gated based on forward and side scatter. The live gate is further analyzed for cell surface expression of Thy1.1 and CD4, taking only the Thy1.1^+^CD4^+^ (donor T cells). Intracellular Foxp3^+^CD4^+^ is then determined from this gated population. (D) Representative CFSE dye dilution three days after transplantation among CFSE‐labeled CD4^+^ T cells recovered from mLN (left) or Spl (right) of mice treated either with DMAG (solid line) or DMSO (grey background). Bottom: Summary of the proliferation indices among donor CD4^+^ T cells isolated from mLN (*n* = 6) or Spl (*n* = 5) (two‐tailed unpaired student's t‐test). (E) Percentages of AnnexinV^+^ (AnnV^+^) cells among donor CD4^+^ T cells in mLN (*n* = 11), Spl (*n* = 11), and liver (*n* = 3) three or seven days after transplantation (one‐tailed unpaired student's *t*‐test). (F) Percentages of CD4^+^ Foxp3^+^ Tregs among donor cells in mLN (*n* = 9), Spl (*n* = 8–9) and liver (*n* = 5–7) seven days after transplantation (two‐tailed unpaired student's *t*‐test). (E) and (F): Pooled data from recipients of either 5 × 10^5^ or 5 × 10^4^ donor CD4^+^ T cells. *P < .05.

To elucidate the mechanism underlying partial protection from aGvHD by Hsp90 inhibition, we performed short‐term experiments analyzing donor CD4^+^ T cell numbers and subset composition in mesenteric lymph nodes (mLN), spleen (Spl) and liver of recipient mice seven days after allogeneic CD4^+^ T cell transplantation. We recovered lower absolute numbers of donor CD4^+^ T cells in mLN of recipient mice treated with DMAG compared to control treated mice when mice had received 5 × 10^5^ (Fig. 2A), by trend also after transplantation of 5 × 10^4^ (Fig. 2B), donor CD4^+^ T cells. Consistent with the differences in the numbers of transplanted CD4^+^ T cells we recovered higher absolute numbers of donor CD4^+^ T cells from mice which had received 5 × 10^5^ (Fig. 2A) versus 5 × 10^4^ CD4^+^ T cells (Fig. 2B). Reduced accumulation of donor CD4^+^ T cells in response to Hsp90 inhibition might be a consequence of reduced proliferation of the CD4^+^ donor T cells. Therefore, we transferred CFSE‐labeled CD4^+^ T cells from C57BL/6 mice into BALB/c recipient mice and analyzed CFSE dye dilution three days after transplantation. We observed similar proliferation of alloreactive T cells in both groups as indicated by the CFSE dilution profiles and the proliferation index of the donor T cells (Fig. 2D). However, the accumulation of CFSE^low^ cells was reduced in the DMAG group (Fig. 2D) suggesting increased apoptosis of the alloreactive CD4^+^ T cells upon Hsp90 inhibition. Indeed, we detected higher frequencies of AnnexinV^+^ cells among donor CD4^+^ T cells isolated from mLN of recipient mice (Fig. 2E). By trend this was also the case in Spl and livers of the recipients (Fig. 2E). Further analysis of the composition of the donor CD4^+^ T cells retrieved on day 7 by flow cytometry revealed that Hsp90 inhibition selectively increased the frequencies of Foxp3^+^ cells among CD4^+^ donor T cells in mLN, but not Spl and liver (Fig. 2F). The relative increase in Treg frequencies in mLN upon Hsp90 inhibition was, thus, accompanied by decreased accumulation of total donor CD4^+^ T cells due to induction of apoptosis in the donor T cells.

### CD4^+^ and CD8^+^ T cell‐mediated aGvHD as well as the Graft versus Tumor (GvT) effect are resistant to Hsp90 inhibition

**Figure 3:**
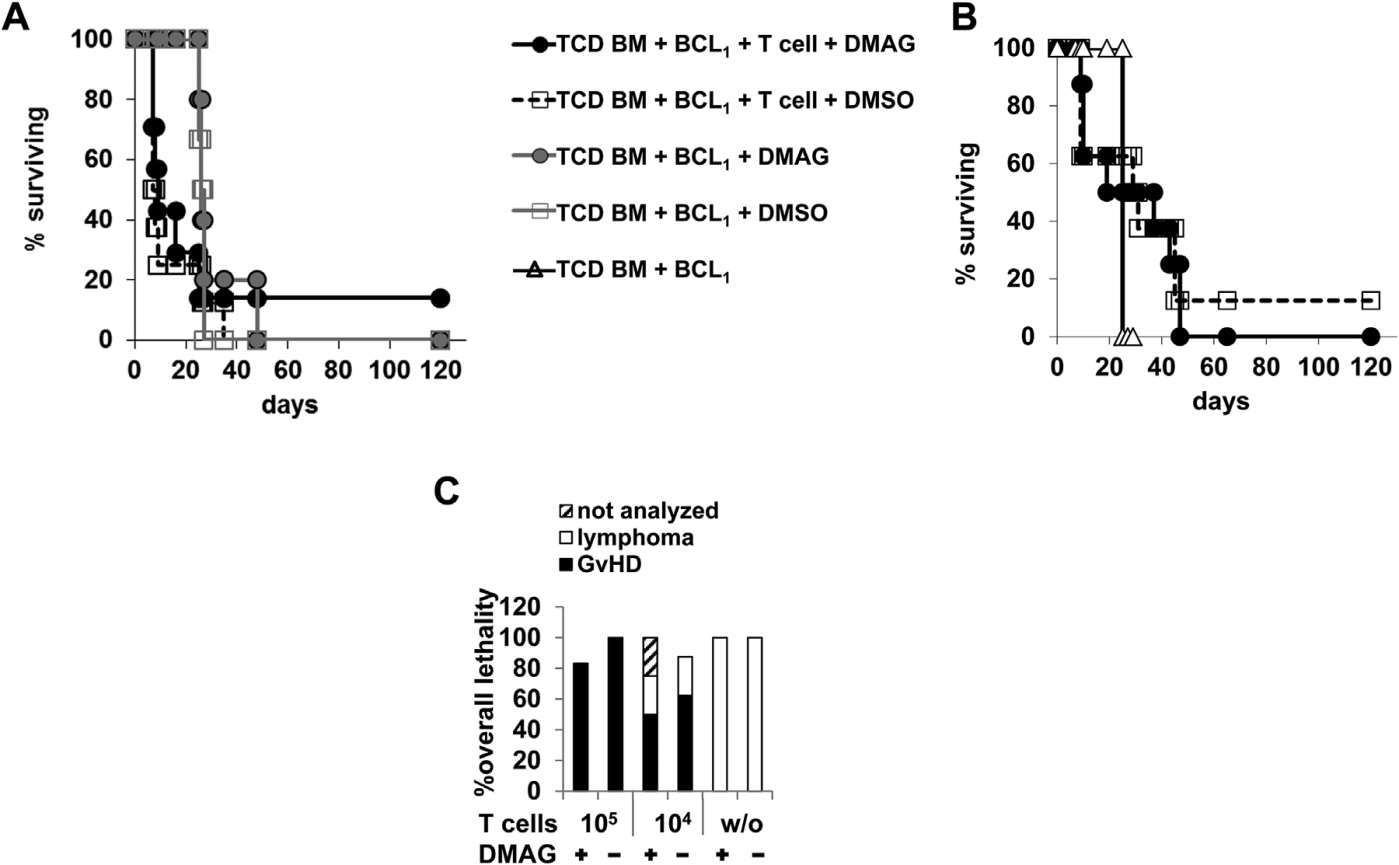
aGvHD induced by CD4^+^ and CD8^+^ T cells is resistant to DMAG‐mediated Hsp90 inhibition in vivo. Lethally irradiated BALB/c mice were reconstituted with 10^7^ C57BL/6 TCD BM cells either alone (*n* = 2) or together with 5 × 10^5^ (A, C) (DMAG *n* = 7, DMSO *n* = 8) or 5 × 10^4^ (B, C) donor T cells (*n* = 8). After TCD BM and total CD4^+^ and CD8^+^ T cell transfer, mice were again treated as in the experiments depicted in Figure 1. (A, B) The percentages of animals surviving over time are depicted. (C) Contribution of different causes of death (lymphoma, aGvHD, not analyzed) to overall lethality. Lymphoma burden was determined by measuring frequencies and absolute numbers of BCL_1_ cells in spleens of recipient mice post mortem (*n* = 4–8). Data from two individual experiments were pooled.

To determine whether Hsp90 inhibition by DMAG would also protect from aGvHD induced by total CD4^+^ and CD8^+^ T cells and to assess its impact on the GvT effect, we inoculated BALB/c mice with BCL_1_ lymphoma cells followed by transplantation of either TCD BM cells only or TCD BM cells together with 5 × 10^5^ (Fig. 3A, C) or 5 × 10^4^ T cells/mouse (Fig. 3B, C). The recipient mice were then treated with DMAG or DMSO as described above. In contrast to CD4^+^ T cell‐induced aGvHD, survival (Fig. 3A, B) and clinical scoring (Suppl. Fig. S1E, SF) during the first 20 days after transplantation of total T cells suggested that DMAG treatment had no protective effect under these conditions regardless of the amount of total T cells transplanted. After day 20 the mice receiving TCD BM cells only showed signs of advanced BCL_1_ lymphoma with, however, no improvement of survival after DMAG treatment. Transplantation of 5 × 10^5^ T cells even worsened overall survival as these mice succumb to aGvHD earlier than the mice suffering from the BCL_1_ lymphoma (Fig. 3A, C). Recipients of 5 × 10^4^ T cells showed better survival than all other groups of mice, but still, with one exception in the DMSO group, all mice died by day 50 post transplantation (Fig. 3B) from either aGvHD or the BCL_1_ lymphoma (Fig. 3C). The frequencies of aGvHD‐ versus BCL_1_ lymphoma‐related lethality were equally high in mice receiving DMAG or DMSO (Fig. 3C). Therefore, DMAG treatment did not ameliorate aGvHD induced by CD4^+^ and CD8^+^ T cells, but also did not negatively interfere with the GvT effect.

### Hsp90 inhibition predominantly targets conventional CD4^+^ T cells as compared to CD8^+^ T cells and Tregs

**Figure 4:**
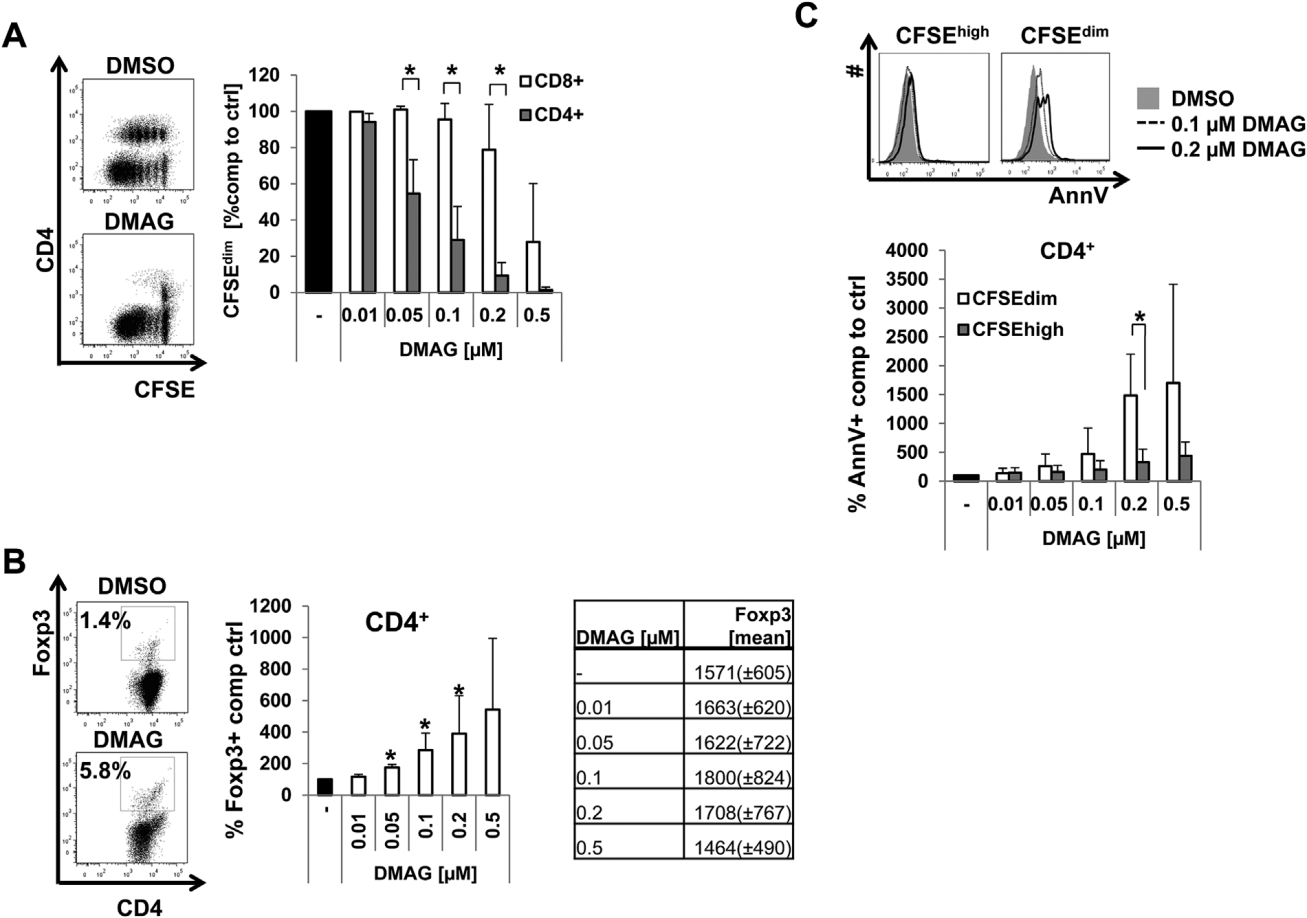
Hsp90 inhibition preferentially targets proliferating CD4^+^ T cells compared to CD8^+^ T cells in vitro. (A‐C) Whole LN cells from C57BL/6 mice were stained with CFSE, stimulated with 0.5 μg/mL anti‐CD3 mAb and 30 U/mL IL‐2 for three days in the presence of the indicated concentrations of DMAG or DMSO as control. Then, CFSE dilution, CD4, CD8, AnnV and intracellular Foxp3 expression were determined using flow cytometry as described in Material and Methods. (A) Left: Representative data of CFSE dilution of stimulated LN cells treated with DMSO ctrl or 0.1 μM DMAG. Right: Percentages of CFSE^dim^ cells among CD4^+^ and CD8^+^ T cells compared to DMSO ctrl (set as 100%). (B) Left: Representative data of Foxp3 expression in CD4^+^ T cells of stimulated LN cells treated with DMSO ctrl or 0.1 μM DMAG. Numbers indicate percentage of Foxp3^+^ cells among CD4^+^ T cells. Middle: Frequencies of Foxp3‐expressing cells among CD4^+^ T cells compared to DMSO ctrl (set as 100%). Right: The table summarizes the mean fluorescence intensities of the Foxp3 staining (mean ± SD) among Tregs after treatment with the indicated DMAG concentrations or DMSO ctrl. (C) Top: Representative data showing AnnexinV binding to CFSE^high^ (left) and CFSE^dim^ (right) CD4^+^ T cells. Bottom: Percentages of AnnV^+^ cells among CFSE^dim^ and CFSE^high^ CD4^+^ T cells. A two‐tailed Mann–Whitney test was used: *P < .05.

Our previous results suggested that Hsp90 blockade mitigates CD4^+^ donor T cell‐mediated aGvHD with no effect on total T cell‐mediated aGvHD in vivo. To identify the cause for this difference we performed in vitro experiments using LN cells of C57BL/6 mice activated by anti‐CD3 mAb/IL‐2 stimulation for three days in the presence of different DMAG concentrations. As shown in Figure 4A, the analysis of proliferation in a CFSE dilution assay demonstrated that Hsp90 inhibition preferentially inhibited CD4^+^ T cells over CD8^+^ T cells, resulting in a significantly decreased CD4^+^/CD8^+^ T cell ratio at the end of the experiment (Suppl. Fig. S2A). As already observed in vivo, we also measured increased percentages of Foxp3^+^ cells among CD4^+^ T cells in response to Hsp90 blockade in vitro (Fig. 4B), confirming a higher susceptibility of conventional CD4^+^ T cells compared to Tregs to Hsp90 inhibition. Analysis of the expression level of Foxp3 by Tregs after DMAG treatment (Fig. 4B) suggests that DMAG is capable of increasing Treg frequencies among CD4^+^ T cells without, however, changing their suppressive activity on a per‐cell basis 29. In order to clarify the underlying mechanisms for the reduced frequencies of proliferating cells among CD4^+^ and CD8^+^ T cells in the presence of DMAG, we measured the proportion of apoptotic cells in anti‐CD3 mAb/IL‐2 activated T cells. Here we found that DMAG induced apoptosis mostly in proliferating (CFSE^dim^) compared to non‐divided (CFSE^high^) CD4^+^ and CD8^+^ T cells (Fig. 4C and Suppl. Fig. S2B). However, there was only a trend toward more apoptosis in CD4^+^ Foxp3^−^ T cells compared to CD4^+^ Foxp3^+^ T cells and in CFSE^dim^ CD4^+^ T cells compared to CFSE^dim^ CD8^+^ T cells upon Hsp90 blockade (Suppl. Fig. S2C and SD), suggesting that under these conditions in vitro growth inhibition dominated over apoptosis induction. Together the activation of total T cells in vitro in the presence of DMAG corroborated the higher sensitivity of conventional CD4^+^ T cells to this drug as compared to Tregs and CD8^+^ T cells.

## Discussion

In this study we addressed the impact of Hsp90 inhibition on aGvHD and the GvT effect in a relevant preclinical mouse model. Our data, using DMAG for Hsp90 inhibition in most and AUY to confirm the data in some experiments, show that blocking Hsp90 partially protected the recipient mice from aGvHD without abrogating the GvT effect. Similar observations have recently been reported in the same mouse aGvHD model using 17‐AAG for Hsp90 inhibition 25. Our observations and those made using 17‐AAG suggest that Hsp90 inhibition might, indeed, constitute a novel approach for the prevention of aGvHD in humans.

In the study using 17‐AAG the authors have shown a direct protective effect on the stem cell niche in the small intestine of which Paneth cells are an important component 25. Enhancing the recovery of the gut epithelium by Hsp90 blockade does, however, not preclude that 17‐AAG or DMAG may also or primarily modulate the function of allogeneic T cells in vivo. In particular, as there are no data so far regarding the impact of Hsp90 inhibition on the integrity of the large intestine which is by far the richer source of microbial products fueling aGvHD 30 as compared to the small intestine 31. We, indeed, observed reduced expansion of allogeneic donor T cells in the mesenteric lymph nodes of the recipient mice which directly drain the gut (Fig. 2A, B). Therefore, protection from aGvHD by DMAG‐mediated Hsp90 blockade was associated with an inhibition of the allogeneic T cell response to which we believe the well documented inhibitory effects of Hsp90 blockade on the T cells themselves 24 have substantially contributed.

The anti‐inflammatory effect of Hsp90 inhibition in the aGvHD model is in line with similar observations in mouse models of autoimmunity involving T cells such as Systemic Lupus Erythematosous (SLE) 32, Multiple Sclerosis 33, Epidermolyis bullosa acquisita 34, and Inflammatory Bowel Disease 35, 36. In the models coming closest to aGvHD, that is, those for Inflammatory Bowel Disease, protection from disease was associated with increased Treg frequencies among CD4^+^ T cells 35 as was the case in our study. In the other disease models this was either not analyzed or shown to be not the case (SLE model) 32. Therefore, the increase in Treg frequencies among CD4^+^ T cells might only occur when Hsp90 is inhibited in the context of intestinal inflammation, but future studies are required to address this point.

The differential responses of the CD4^+^ and CD8^+^ T cell subsets studied to Hsp90 inhibition may be explained at the molecular level looking at the client proteins released from Hsp90 upon pharmacological inhibition: Hsp90 client proteins include key signaling molecules like the Akt kinase, the mTOR Complex 1 (mTORC1) component Raptor 37 and crucial transcription factors like Heat Shock Factor 1 (HSF1). In case of the Akt kinase and Raptor the release from Hsp90 leads to their degradation. Therefore, cells highly depending on Akt/mTORC1 signaling for proliferation and survival like CD4^+^ conventional T cells are more affected by Akt degradation than Tregs 38. Similarly, it has been shown that not only Tregs but also (human) CD8^+^ T cells may proliferate in the presence of the mTOR inhibitor rapamycin 39, showing that CD8^+^ T cells as well as Tregs do not require this molecular axis for activation. Moreover, rapamycin treatment may even enhance CD8^+^ T cell responses, in particular memory formation 40. In contrast to Akt, the release of HSF1 from Hsp90 leads to its translocation to the nucleus and the initiation of target gene transcription. Among others HSF1 induces expression of Hsp70 which physically interacts with Foxp3 and increases the suppressive activity of Tregs 41. Thus, reduced dependency on the Akt‐mTOR axis 38 and increased HSF1‐mediated transcription sufficiently explain why Tregs and CD8^+^ T cells are less affected by Hsp90 inhibition than conventional CD4^+^ T cells.

In contrast to the studies carried out in models of autoimmunity in which therapeutic success means abrogation of the pathological immune response, our study also addressed the impact of Hsp90 inhibition on protective immunity, that is, in the case of allogeneic HSC transplantation the GvT effect. In the model we used the GvT effect is mediated by CD8^+^ T cells 42. Our data show that Hsp90 inhibition was compatible with killing by CD8^+^ T cells in vitro (Suppl. Fig. S3) and anti‐tumor activity of the CD8^+^ T cells in vivo (Fig. 3), but aGvHD induced by CD4^+^ and CD8^+^ T cells was also not ameliorated. This means that Hsp90 inhibition alone was not sufficient to separate aGvHD suppression from maintenance of the GvT effect in the mouse model. Here, the poor response of the CD8^+^ T cells to Hsp90 inhibition alone certainly contributed to this effect. As the addition of a PI3K inhibitor has previously been shown to overcome rapamycin resistance in human CD8^+^ T cells 43 we assume that combining Hsp90 blockade with PI3K inhibition might also protect mice from aGvHD pathology induced by CD8^+^ T cells and may also be efficacious in humans. Indeed, we recently demonstrated that combination of Hsp90 and PI3K inhibition targets proliferation of alloreactive CD4^+^ as well as CD8^+^ T cells 44.

Although Hsp90 inhibitors were developed as bona fide anti‐cancer drugs, the BCL_1_ lymphoma cells, which we used to study the GvT effect in our model, did not respond to the short‐term Hsp90 inhibitor treatment we applied to the mice in order to modulate the allogeneic T cell response in the recipient. This might, however, be different in patients treated, for example, for chronic myelogenous leukemia (CML), and to a lesser degree acute myelogenous leukemia (AML) or multiple myeloma (MM), for which a remission‐inducing GvT effect can frequently be observed 4. Here, Hsp90 inhibition after allogeneic HSC transplantation might directly target the CML 45, 46, AML 47 or MM cells 48, thus synergizing with the GvT effect of the transplanted T cells.

In summary, our study shows that Hsp90 inhibition may be used to protect recipients of allogeneic T cell grafts from aGvHD. With respect to the GvT effect Hsp90 inhibition did not abrogate the anti‐lymphoma activity of the transplanted CD8^+^ T cells. However, combination therapy of Hsp90 with, for example, PI3K inhibition might ameliorate CD8^+^ T cell‐induced aGvHD pathology, hopefully maintaining GvT activity. In patients, synergy between a direct anti‐cancer and the GvT effect may greatly increase the efficacy of Hsp90 inhibition. Therefore, we do hope that our data from the mouse model will help to further evaluate the use of Hsp90 inhibitors for the prophylaxis/ treatment of aGvHD in humans.

### Conflict of Interest

None declared.

## Supporting information

Additional supporting information may be found in the online version of this article at the publisher's web‐site.

**Figure S1**. Treatment with the Hsp90 inhibitor AUY in vivo attenuates CD4^+^ T cell‐mediated aGvHD.
**Figure S2**. Hsp90 preferentially targets proliferating CD4^+^ T cells compared to CD8^+^ T cells in vitro.
**Figure S3**. DMAG‐mediated inhibition of Hsp90 leads to a dose‐dependent reduction in CTL activity in vitro.
