# Supplementary material for: Hsp90 inhibition ameliorates CD4+ T cell‐mediated acute Graft versus Host disease in mice
Source: Immun Inflamm Dis. 2016 Oct 10;4(4):463–73. doi: 10.1002/iid3.127 (PMC5134726; doi:10.1002/iid3.127)
Supplement: Supplementary file 1 — Figure S1. Treatment with the Hsp90 inhibitor AUY in vivo attenuates CD4+ T cell‐mediated aGvHD. Figure S2. Hsp90 preferentially targets proliferating CD4+ T cells compared to CD8+ T cells in vitro. Figure S3. DMAG‐mediated inhibition of Hsp90 leads to a dose‐dependent reduction in CTL activity in vitro. [file IID3-4-463-s001.pdf]

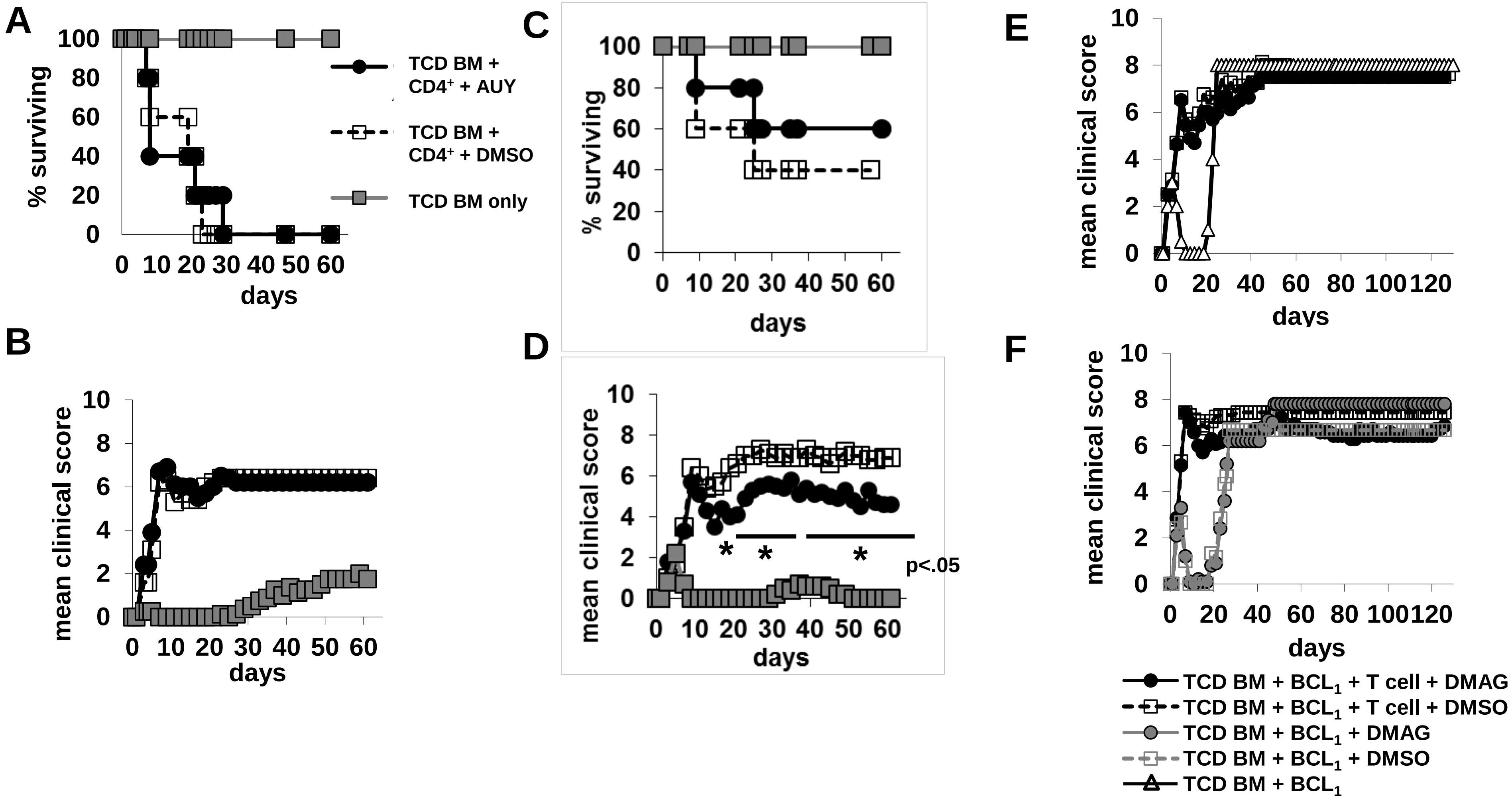

Suppl. Figure 1. Treatment with the Hsp90 inhibitor AUY *in vivo* attenuates $CD4^+$ T cell-mediated aGvHD. Lethally irradiated BALB/c mice were reconstituted with 10^7 C57BL/6 TCD BM cells either alone (n=5) or with $5x10^5$ (A, B) or $5x10^4$ (C, D) donor $CD4^+$ T cells (n=4-5), respectively. After TCD BM and $CD4^+$ T cell transfer, mice received 50 µg NVP-AUY922 (AUY)/day or solvent only (DMSO) both diluted in PBS/TWEEN from days 0 to +2 post-BM transplantation. The percentages of animals surviving over time and (A, C) and the mean clinical scores of recipient animals (B, D) are depicted (one-tailed Mann-Whitney test).Find legend for A-D on the right of graph A.

(E,F) aGvHD induced by $CD4^+$ and $CD8^+$ T cells is resistant to DMAG-mediated Hsp90 inhibition *in vivo*. This part of the figure shows the clinical scores of the animals for which survival curves are depicted in Figure 3. (E) Mean clinical scores of lethally irradiated BALB/c mice reconstituted with 10^7 C57BL/6 TCD BM cells either alone (n=2) or together with $5x10^4$ (n=8) donor T cells or (F) $5x10^5$ (DMAG n=7, DMSO n=8). Data from two individual experiments were pooled. Find legend for E and F below graph F.

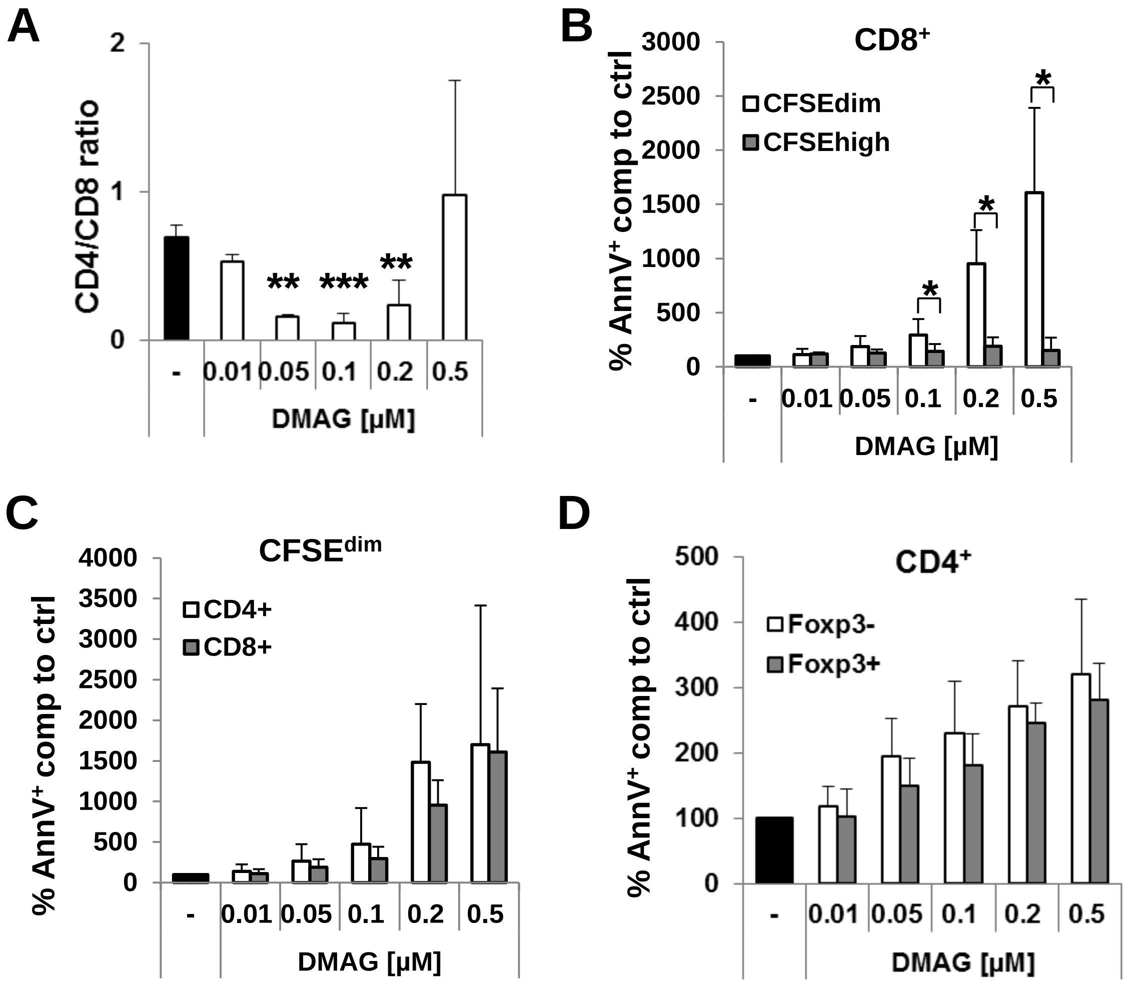

Suppl. Figure 2. Hsp90 preferentially targets proliferating CD4$^+$ T cells compared to CD8$^+$ T cells *in vitro.*

Here, additional information is provided regarding the data shown in Figure 4. (A-D) Whole LN cells from C57BL/6 mice were stained with CFSE, stimulated with 0.5 µg/ml anti-CD3 mAb and 30 U/ml IL-2 for 3 days in the presence of the indicated concentrations of DMAG or DMSO as control. Then, CFSE dilution, CD4, CD8, AnnV and intracellular Foxp3 expression were determined using flow cytometry as described in Material and Methods. (A) CD4/CD8 ratios after three days of *in vitro* culture in the presence of the indicated amounts of DMAG or DMSO as ctrl. (B) Percentages of AnnV$^+$ cells among Foxp3$^+$ and Foxp3$^-$ CD4$^+$ T cells. (C) Left: Percentages of AnnV$^+$ cells among CFSE^{high} and CFSE^{dim} CD8$^+$ T cells compared to DMSO ctrl (set as 100%). Right: Representative AnnV stainings of CFSE^{high} and CFSE^{dim} CD8$^+$ T cells. (D) Percentages of AnnV$^+$ cells among CFSE^{dim} CD4$^+$ and CD8$^+$ T cells compared to DMSO ctrl (set as 100%). Data are given as mean values + standard deviation of five independent experiments. A paired two-tailed student's t-test was used: * $p < .05$, ** $p < .01$, *** $p < .001$.

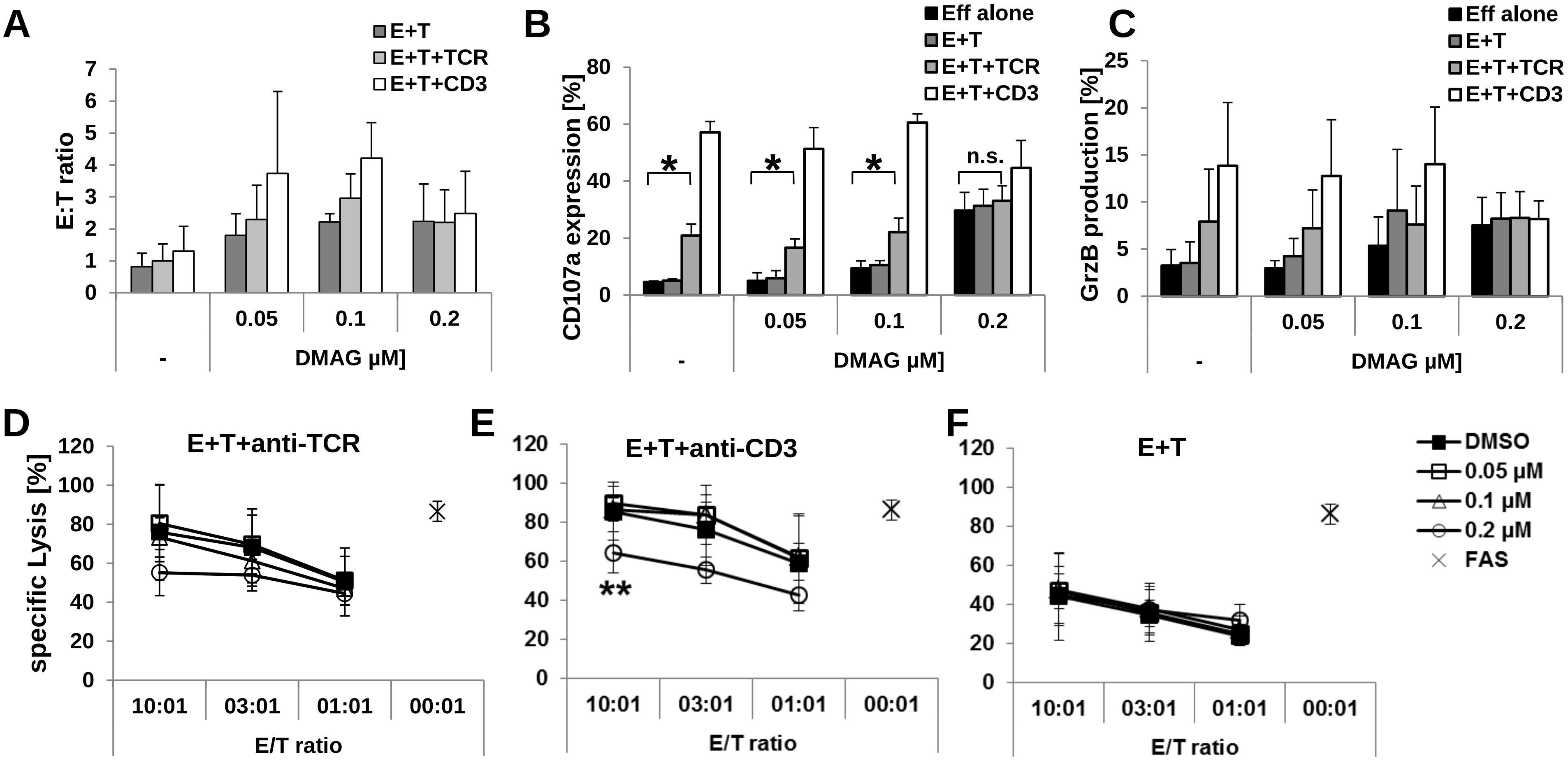

Suppl. Figure 3. DMAG-mediated inhibition of Hsp90 leads to a dose-dependent reduction in CTL activity *in vitro*. To assess the effect of Hsp90 inhibition on the cytotoxic potential and degranulation activity of $CD8^+$ T cells, we used 'Con A blasts', i.e. isolated LN cells were activated with Concanvalin A (5 µg/ml, Sigma-Aldrich) for 3 days in the presence of different concentrations of DMAG or DMSO ctrl. Con A blasts were co-incubated with CFSE-labeled A20J target cells in a 96 well V-bottom plate at different effector to target ratios for two or four hours. To trigger re-directed lysis either anti-CD3 mAb or anti-β TCR mAb H57-H7 (BD) were added at a final concentration of 10 µg/ml. To assess the susceptibility of A20J cells towards apoptotic stimuli, they were incubated with the anti-CD95 mAb Jo2 (BD) at a final concentration of 0.1 µg/ml. After the killing period, viability of the A20J target cells was determined by first counter-staining the cells with propidium iodide and AnnexinV APC (BD) followed by FACS analysis. The degranulation activity of $CD8^+$ Con A blasts was measured by adding the anti-CD107a mAb to the co-culture of Con A blasts and A20J cells followed by surface staining for CD8a and subsequently intracellular staining for Granzyme B expression.

(A) E:T ratio, (B) CD107a and (C) intracellular GrzB expression by $CD8^+$ Con A blasts at the end of the culturing period. (D-F) Cytolytic activity of the Con A blasts. Data are given as mean values ± standard deviation of at least four independent experiments. A two-tailed paired student's t-test was used: * $p < .05$, ** $p < .01$.
